# Inhibition of the NAD-Dependent Protein Deacetylase SIRT2 Induces Granulocytic Differentiation in Human Leukemia Cells

**DOI:** 10.1371/journal.pone.0057633

**Published:** 2013-02-27

**Authors:** Yoshitaka Sunami, Marito Araki, Yumi Hironaka, Soji Morishita, Masaki Kobayashi, Ei Leen Liew, Yoko Edahiro, Miyuki Tsutsui, Akimichi Ohsaka, Norio Komatsu

**Affiliations:** 1 Department of Hematology, Juntendo University School of Medicine, Tokyo, Japan; 2 Department of Transfusion Medicine and Stem Cell Regulation, Juntendo University School of Medicine, Tokyo, Japan; 3 Central Research Laboratories, Sysmex Corporation, Hyogo, Japan; 4 Fujii Memorial Research Institute, Otsuka Pharmaceutical Co., Ltd., Shiga, Japan; Roswell Park Cancer Institute, United States of America

## Abstract

Sirtuins, NAD-dependent protein deacetylases, play important roles in cellular functions such as metabolism and differentiation. Whether sirtuins function in tumorigenesis is still controversial, but sirtuins are aberrantly expressed in tumors, which may keep cancerous cells undifferentiated. Therefore, we investigated whether the inhibition of sirtuin family proteins induces cellular differentiation in leukemic cells. The sirtuin inhibitors tenovin-6 and BML-266 induce granulocytic differentiation in the acute promyelocytic leukemia (APL) cell line NB4. This differentiation is likely caused by an inhibition of SIRT2 deacetylase activity, judging from the accumulation of acetylated α-tubulin, a major SIRT2 substrate. Unlike the clinically used differentiation inducer all-*trans* retinoic acid, tenovin-6 shows limited effects on promyelocytic leukemia–retinoic acid receptor α (PML-RAR-α) stability and promyelocytic leukemia nuclear body formation in NB4 cells, suggesting that tenovin-6 does not directly target PML-RAR-α activity. In agreement with this, tenovin-6 induces cellular differentiation in the non-APL cell line HL-60, where PML-RAR-α does not exist. Knocking down SIRT2 by shRNA induces granulocytic differentiation in NB4 cells, which demonstrates that the inhibition of SIRT2 activity is sufficient to induce cell differentiation in NB4 cells. The overexpression of SIRT2 in NB4 cells decreases the level of granulocytic differentiation induced by tenovin-6, which indicates that tenovin-6 induces granulocytic differentiation by inhibiting SIRT2 activity. Taken together, our data suggest that targeting SIRT2 is a viable strategy to induce leukemic cell differentiation.

## Introduction

Cancerous cells are generally undifferentiated, due in part to a loss of function of differentiation-regulatory elements resulting from aberrant gene expression. Targeting the system that keeps cancerous cells undifferentiated is a logical strategy to induce terminal differentiation and subsequent cell proliferation arrest and/or apoptosis. To achieve this goal, it is important to identify molecular targets that regulate cellular differentiation.

Thus far, all-*trans* retinoic acid (ATRA) is the only differentiating agent used in the clinic, being part of the standard treatment of acute promyelocytic leukemia (APL) [1]. In APL cells in 90% of APL cases, retinoic acid receptor α (RAR-α) and its partner promyelocytic leukemia (PML) or other proteins are fused due to chromosomal rearrangement [2]. This PML-RAR-α fusion protein plays a causal role during leukemia development in mouse models [3]. The mechanistic models of how PML-RAR-α promotes leukemogenesis are as follows [3], [4]: (a) PML-RAR-α fusion protein binds to the transcriptional regulatory sequences of RAR-α target genes and recruits co-repressors to block the normal RAR-α function required for granulocytic differentiation; and (b) by interfering with the multimerization of PML proteins, PML-RAR-α blocks the formation of PML nuclear bodies (NBs) that seem to be required for granulocytic differentiation through the regulation of gene expression and protein degradation.

Upon ATRA treatment, ATRA directly binds to the RAR-α moiety, induces the conformational change of PML-RAR-α to dissociate from the co-repressor, and simultaneously activates RAR-α function to induce granulocytic differentiation in APL cells [3]. ATRA treatment also promotes the degradation of PML-RAR-α by 2 independent protein-degradation pathways: the ubiquitin-proteasome [5] and the autophagy system [6]. PML-RAR-α degradation represses the accumulation of PML-RAR-α oncogene products in leukemia cells and subsequently promotes PML-NB formation in APL cells.

Because abnormal recruitment of histone-deacetylases (HDACs) by PML-RAR-α is a key mechanism of the pathogenesis of APL [3], targeting HDAC to differentiate APL cells using small molecules has been extensively studied. Although HDAC inhibitors are strongly cytotoxic against APL cells[7]–[9] and other cancerous cells [10]–[12], they exhibit a limited potential for inducing cellular differentiation in APL cells [7], [9], [13], [14]. This evidence suggests that although aberrant recruitment of the HDAC complex by PML-RAR-α represents a relevant pathogenetic mechanism, inhibition of the enzymatic activity of the complex is not sufficient to restore the differentiation potential of APL cells [15].

The human sirtuin family, SIRT1 to SIRT7, possesses a unique NAD-dependent protein deacetylase activity and plays diverse roles in cells, including the regulation of DNA repair, cell cycle, metabolism, and cell survival [16], [17]. Sirtuin localization is also diverse and includes the nucleus, cytosol, and mitochondria. [16] Nuclear-localized SIRT1, SIRT2, SIRT6, and SIRT7 regulate the activities of transcription factors through direct deacetylation. In addition, even cytosolic-localized SIRT1 and SIRT2 control the transcriptional program by regulating the localization of transcription factors by deacetylation, which has been well characterized in the SIRT-FOXO axis [18], [19].

In tumorigenesis, the roles of sirtuins are complicated due to their wide range of substrates and cellular functions [16], [20]. SIRT1 is expressed at a higher level in cancerous cells and promotes oncogenesis by suppressing p53 activity through deacetylation of lysine 382 [21], [22]. However, in a colon cancer mouse model, increased *SIRT1* expression suppresses cell proliferation and tumor formation [23]. Another study reported that *SIRT1* haplo-insufficiency promotes tumorigenesis in mice, implying that *SIRT1* is a tumor suppressor [24]. Thus, SIRT1 has a dual role in tumorigenesis whose manifestation may depend on the specific cell, tumor type, stage, or differentiation level [16]. While *SIRT2* down-regulation induces apoptosis [25], reduced expression of *SIRT2* in human cancer cells have been reported [26], [27]. In rodents, *SIRT2* deficiency induces chromosome alterations and subsequent tumor development, defining SIRT2 as a tumor suppressor [27]. SIRT6 is involved in DNA repair and metabolism and possess an apparent tumor suppressor role [28]. Overexpression of SIRT6 induces massive apoptosis in cancer cells [29]. In addition to the abovementioned sirtuins, the involvement of mitochondrial SIRT3-5 and nuclear SIRT7 in tumorigenesis still remains unclear [16]. Despite lacking a clear understanding of sirtuin activities in tumorigenesis, some sirtuin inhibitors exhibit strong cytotoxicity against cancerous cells and show promising anti-cancer effects in mouse models [22].

SIRT1 is involved in granulocyte colony-stimulating factor-induced myeloid differentiation in normal CD34^+^ cells [30]. Accordingly, we hypothesized that sirtuin de-regulation plays a role in keeping leukemic cells undifferentiated and that inhibition of sirtuin activity would induce cellular differentiation in leukemic cells. Although a number of HDAC inhibitors have been examined as potential differentiation-inducing compounds in leukemia cells, those pan-HDAC inhibitors do not efficiently block the enzymatic activity of sirtuins [22]. In addition, the SIRT2 inhibitor AC93253 has an anti-proliferative effect on AML (acute myeloid leukemia) cells, but the capacity of this compound to induce cellular differentiation remains elusive [31]. Here, we examined whether sirtuin inhibitors are capable of inducing cellular differentiation in leukemia cells. We subsequently assessed which sirtuins induce granulocytic differentiation in leukemia cells when they are inhibited.

## Results

### Tenovin-6 Inhibits Cell Proliferation and Induces Apoptosis in NB4 Cells

**Figure 1:**
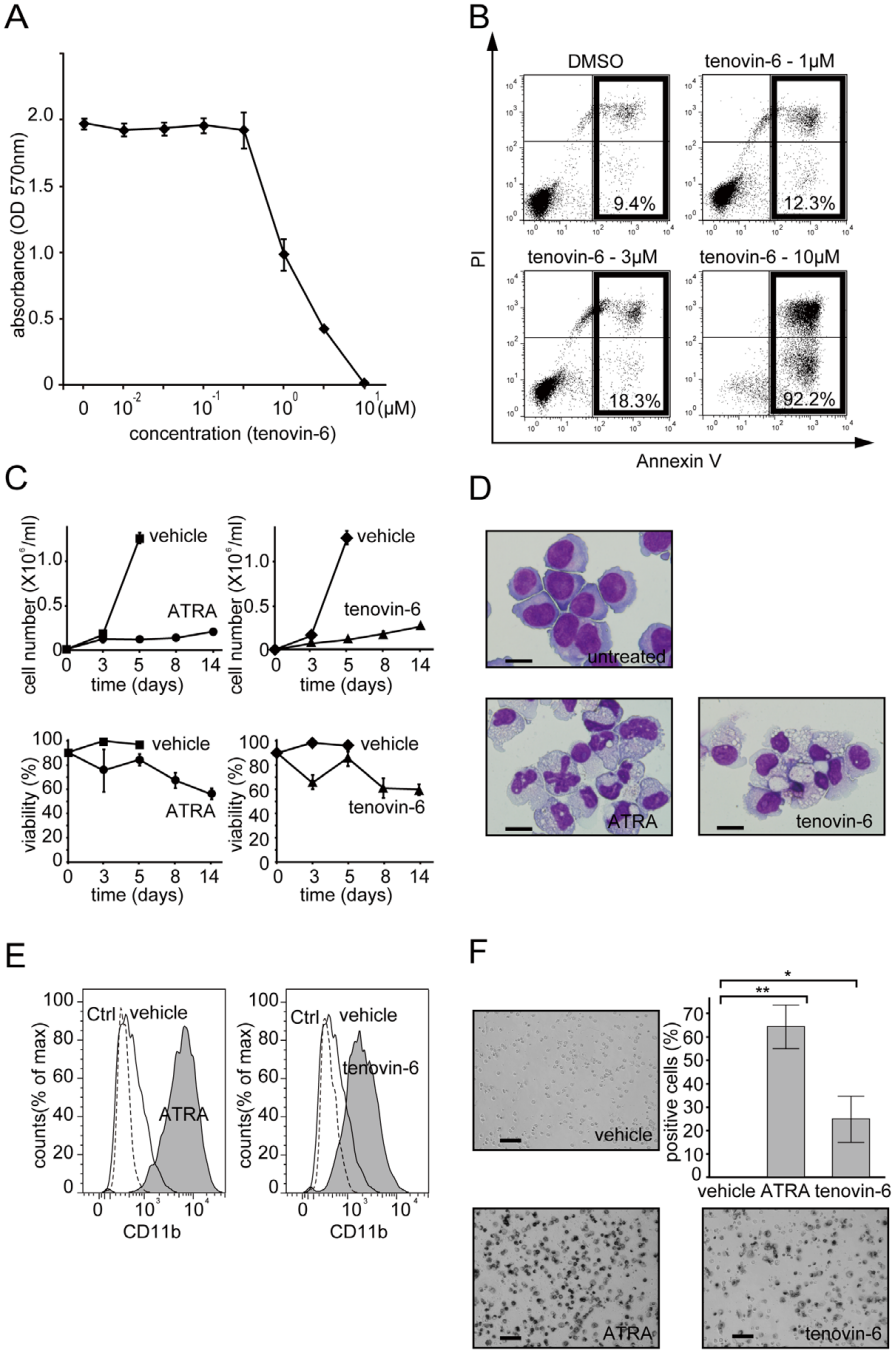
Tenovin-6 inhibits cell proliferation, induces apoptosis, and promotes granulocytic differentiation in NB4 cells. (A) NB4 cells were cultured for 48 hours in the presence of tenovin-6 (0.01, 0.03, 0.1, 0.3, 1, 3, and 10 µM) or vehicle (DMSO). Cell proliferation was monitored by MTT assay. The results are presented as means ± SD of 6 replicates. Representative data are shown. Experiments were performed in triplicate, and the error bars show mean ± SD. (B) Annexin-V/PI assay was performed after 24 hours treatment with the indicated amount of tenovin-6 or DMSO. The population of cells undergoing apoptosis is indicated. Note that despite the strong inhibition of cell proliferation in Figure 1A, the induction of apoptosis by 3 µM tenovin-6 is low. (C) A long-term culture experiment on tenovin-6-treated NB4 cells was performed. Cells were treated with tenovin-6 (3 µM), ATRA (1 µM), DMSO (vehicle for tenovin-6), or ethanol (vehicle for ATRA). Live cells were counted and are depicted in graphs on the top. Dead cell numbers were determined by trypan blue dye inclusion. The calculated viability is depicted in graphs on the bottom. Representative data are shown. Experiments were performed in triplicate, and error bars show mean ± SD. (D) Morphologic alternation was examined 7 days after treatment with 3 µM of tenovin-6 or 1 µM of ATRA by Wright-Giemsa staining. Untreated NB4 cells were used as a control. Either tenovin-6- or ATRA-treated NB4 cells presented highly differentiated morphologies (eg, lower nucleus:cytoplasm ratio). Scale bar represents 20 µm. (E) FACS analysis of tenovin-6 (3 µM)- or ATRA (1 µM)-treated NB4 cells (gray shadow) using PE-CD11b. These cells had increased expression levels of CD11b compared to untreated cells (black line). The PE-IgG control profile is drawn as a dashed line. (F) NBT reduction capacity in ATRA-, tenovin-6-, or DMSO-treated NB4 cells was examined. The captured images were taken 5 days after treatment. The black staining indicates that cells possess NBT reduction capacity, one of the signature properties of matured granulocytes. Stained cells counted at day 5 are depicted in the graph. Counting was performed in 3 independent microscope fields that covered at least 200 cells each. Error bars show mean ± SD (n = 3). *P<0.05. **P<0.01.

To examine the effect of a sirtuin inhibitor on APL cell differentiation, we chose tenovin-6, a potent compound against solid tumors in mouse models [32]. We first investigated the cytotoxicity of tenovin-6 against the APL cell line NB4. As reported previously for solid tumors [32] and chronic myeloid leukemia cells [33]–[35], tenovin-6 blocked cell proliferation in a concentration-dependent manner (Figure 1A). At 10 µM, tenovin-6 drastically induced cellular apoptosis after 48 hours of treatment in NB4 cells (Figure 1B), indicating that growth perturbation by tenovin-6 is due to an induction of apoptosis in that time frame. We also observed an induction of apoptosis in a fraction of cells treated with a lower concentration (3 µM) of tenovin-6 (Figure 1B), but the percentage of apoptotic cells (18.3%) was considerably lower than that expected from the growth inhibition rate (79% reduction from the control) (Figure 1A).

### Tenovin-6 Promotes Granulocytic Differentiation in NB4 Cells

To further investigate what happens to NB4 cells when treated with a lower concentration of tenovin-6, we monitored cell viability for 14 days after treatment with 3 µM tenovin-6. As shown in Figure 1C, 3 µM tenovin-6-treated cells maintained their viability at approximately 60% even after 2 weeks of culture, without a significant increase in cell proliferation. Interestingly, 1 µM ATRA-treated cells had virtually the same level of cell viability and cell proliferation profile, which prompted us to investigate whether tenovin-6 induces cellular differentiation in NB4 cells as ATRA does. To examine the cellular morphology of NB4 cells treated with tenovin-6 or ATRA, we prepared cytospin smears and performed Wright-Giemsa staining. A light microscopy examination revealed that although tenovin-6-treated cells showed none of the obvious nuclear phenotypes (such as segmented nuclei) observed in ATRA-treated cells, tenovin-6-treated cells showed a highly differentiated cellular morphology, such as a lower nucleus:cytoplasm ratio (Figure 1D). This observation indicates that the strong growth inhibition resulting from treatment with 3 µM tenovin-6 and the lower ratio of apoptotic cells are likely due to the induction of cell cycle arrest and subsequent cell differentiation.

Fluorescence-activated cell sorting (FACS) analysis revealed that tenovin-6- and ATRA-treated cells showed significantly increased expression levels of CD11b, a surface marker of the granulocytic phenotype, compared to cells cultured with vehicle alone (Figure 1E). To further characterize tenovin-6-treated cells, we performed NBT reduction assays. As shown in Figure 1F, tenovin-6-treated cells exhibited increased NBT reduction capacity, the hallmark of maturation of granulocytes, similar to the positive-control ATRA-treated cells. These findings indicate that tenovin-6 induces granulocytic differentiation in NB4 cells.

### Tenovin-6 has Limited Effects on PML-RAR-α Stability and PML-NB Formation

**Figure 2:**
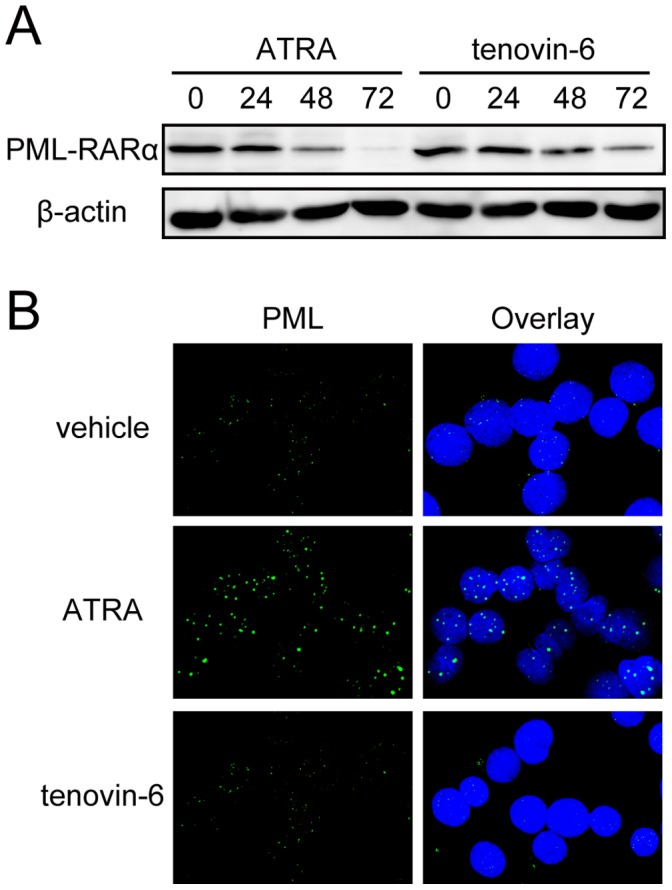
Tenovin-6 has limited effects on PML-RAR -**α stability and PML-NB formation.** (A) PML-RAR-α accumulation was examined when NB4 cells were cultured in the presence of ATRA (1 µM) or tenovin-6 (3 µM) for the indicated time. 175 µg extracts were loaded in each lane. Note that longer incubation with tenovin-6 lowered PML-RAR-α accumulation, but some remained at 72 hours, whereas PML-RAR-α was rapidly degraded in ATRA-treated cells, and the protein had nearly disappeared at 72 hours. We obtained similar results in another, independent experiment. (B) PML-NB formation in tenovin-6 (3 µM)- or ATRA (1 µM)-treated or untreated NB4 cells after 120 hours of incubation was examined by immunofluorescence microscopy using anti-PML antibody. While ATRA treatment induced PML-NB formation, as reported previously [37], [38], tenovin-6 treatment did not show any effect on PML-NB formation.

We next investigated the mechanism by which tenovin-6 induces granulocytic differentiation in NB4 cells. Because PML-RAR-α degradation [5], [36] and subsequent induction of PML-NB formation are critical events for ATRA-induced differentiation of APL cells [37], [38], we performed an immunoblot analysis to monitor PML-RAR-α accumulation after tenovin-6 treatment. PML-RAR-α protein virtually disappeared 72 hours after ATRA treatment, as previously described [5], [36]. In contrast, its expression level decreased but persisted 72 hours after tenovin-6 treatment (Figure 2A). Consistent with this observation, ATRA, but not tenovin-6, induced PML-NB formation in NB4 cells (Figure 2B).

### Tenovin-6 Induces Granulocytic Differentiation in the PML-RAR-α-negative Myeloblastic Leukemia Cell Line HL-60

**Figure 3:**
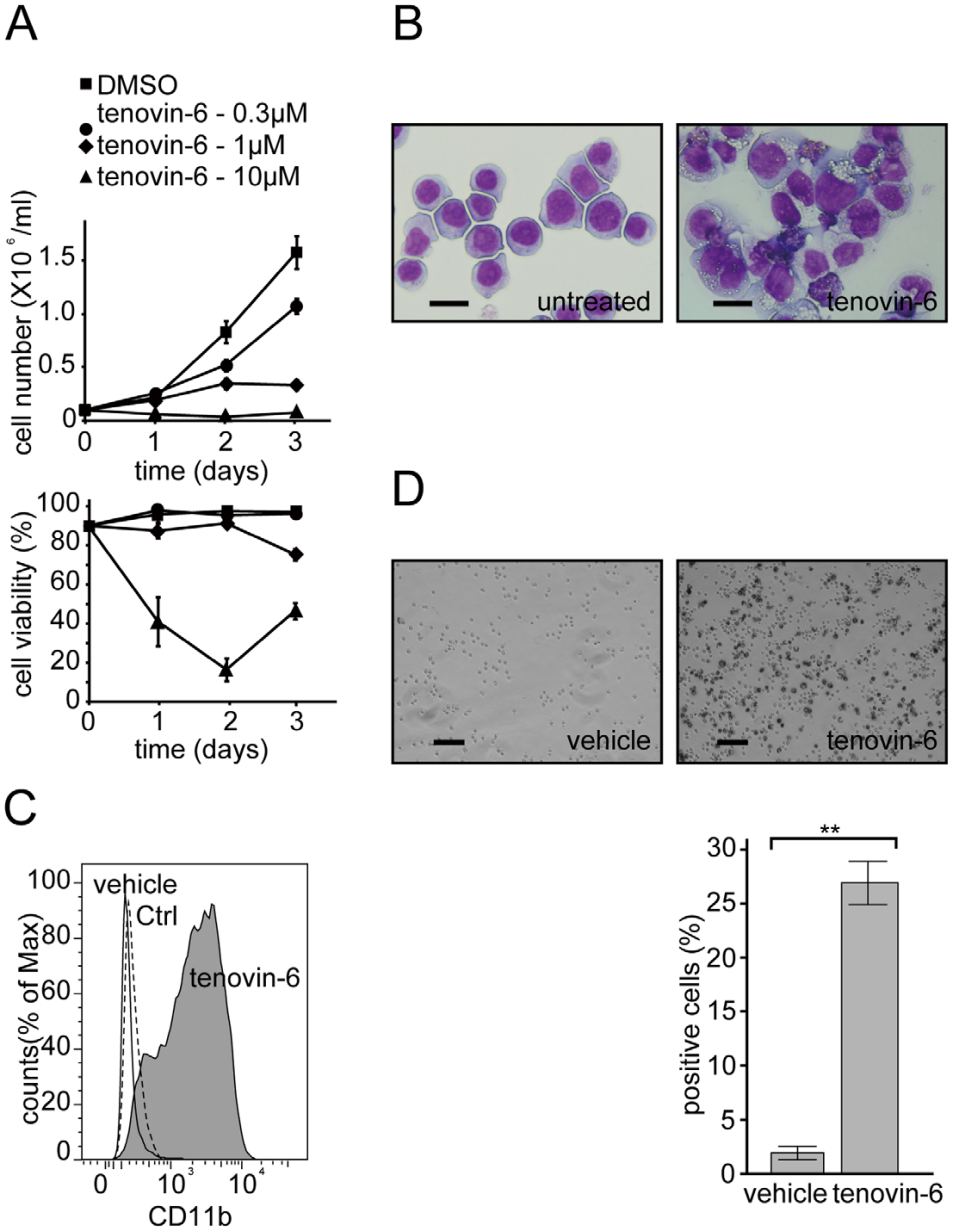
Tenovin-6 induces granulocytic differentiation in the PML-RAR -**α-negative myeloblastic leukemia cell line HL-60.** (A) HL-60 cells were cultured in the presence of the indicated amount of tenovin-6 or DMSO (vehicle). Total viable cell counts (upper graph) and viabilities (lower graph) are depicted. Note that at 1 µM, cell proliferation was strongly inhibited, with relatively high viability (75%). Representative data are shown. Experiments were performed in triplicate, and error bars show mean ± SD. (B) Tenovin-6 (1 µM)-treated HL-60 cells show a more differentiated cell morphology in Wright-Giemsa staining. (C) FACS analysis of tenovin-6 (3 µM)-treated HL-60 cells (gray shadow) using PE-CD11b. The cells demonstrated increased expression levels of CD11b compared with untreated cells (black line). The PE-IgG control profile is drawn as a dashed line. (D) Tenovin-6 (1 µM)-treated HL-60 cells became positive for NBT reduction. Counting was performed as described in Figure 1F. Error bars show mean ± SD (n = 3). **P<0.01.

Tenovin-6 had limited effects on both PML-RAR-α degradation and subsequent PML-NB formation, suggesting that these events are dispensable for tenovin-6-induced granulocytic differentiation of APL cells. Accordingly, we investigated whether tenovin-6 induces granulocytic differentiation in leukemia cells lacking PML-RAR-α. We treated the PML-RAR-α-negative myeloblastic leukemia cell line HL-60 with different doses of tenovin-6 and found that a modest concentration of tenovin-6 (1 µM) blocked cell proliferation while maintaining a high viability score (Figure 3A). At this concentration, HL-60 cells exhibited more differentiated cellular morphology on Wright-Giemsa staining (Figure 3B) and higher expression of the CD11b granulocyte marker (Figure 3C). More than 20% of cells became positive for NBT reduction capacity, implying that tenovin-6 induced at least partial granulocytic differentiation in HL-60 cells (Figure 3D). Taken together, these data suggest that neither PML-RAR-α degradation nor subsequent PML-NB formation is a prerequisite for tenovin-6-induced granulocytic differentiation.

### Tenovin-6 Induces Granulocytic Differentiation in APL Cells via Inhibition of SIRT2 but not SIRT1

**Figure 4:**
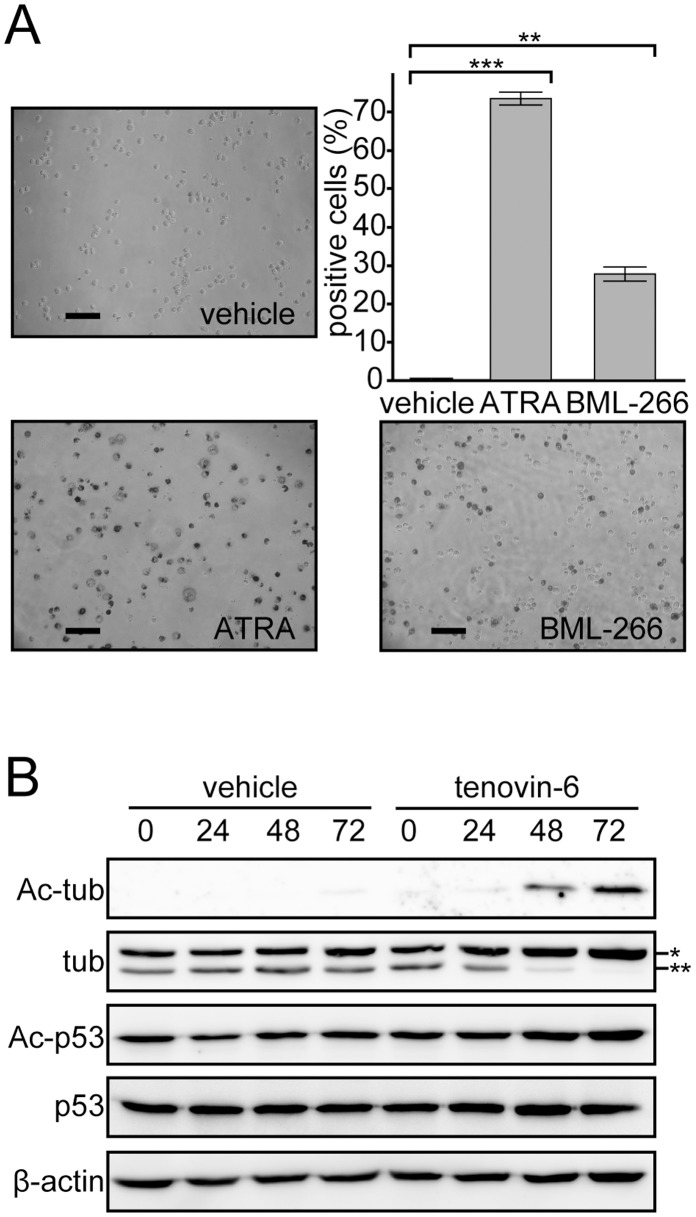
Tenovin-6 induces granulocytic differentiation in APL cells via inhibition of SIRT2, but not of SIRT1. (A) NB4 cells were treated with another sirtuin inhibitor, BML-266 (5 µM), for 3 days, and images from NBT reduction assays are presented. Positive cells in the NBT reduction assay were counted under the microscope as described in Figure 1F and are depicted in the graph. Error bars show mean ± SD (n = 3). **P<0.01. ***P<0.001. (B) Acetylated forms of SIRT1 and SIRT2 substrates p53 and α-tubulin, respectively, were examined after tenovin-6 (3 µM) or DMSO (vehicle) treatment for the indicated period. 42 µg extracts were loaded in each lane. Total tubulin and p53 levels were unchanged. β-Actin immunoblot analysis was performed to obtain a loading control. A single and double asterisk indicates acetylated and unacetylated α-tubulin, respectively.

Next, we investigated whether tenovin-6 inhibits sirtuin family proteins to induce granulocytic differentiation in NB4 cells. First, we examined whether another sirtuin inhibitor, BML-266, showed similar effects as tenovin-6. We determined the concentration of BML-266 that would allow us to monitor cell differentiation as described above. At 5 µM BML-266, NB4 cells maintained high viability (data not shown) and became positive for NBT reduction capacity (Figure 4A), indicating that similar to tenovin-6, BML-266 induce granulocytic differentiation in NB4 cells.

Because 2 independent inhibitors against sirtuin family proteins exhibited the capacity to induce granulocytic differentiation in NB4 cells, we assessed which sirtuin(s) were inhibited at the concentrations that induced cell differentiation by monitoring sirtuin substrate proteins. We decided to examine the involvement of SIRT1 and SIRT2 in tenovin-6-induced granulocytic differentiation because (a) tenovin-6 has a higher IC_50_ against SIRT1 and SIRT2 than other sirtuin family proteins [32] and (b) BML-266 is proposed to be a SIRT2-specific inhibitor, although there are no available data concerning its IC_50_ value against other sirtuins [39].

Although tenovin-6 reportedly induces an accumulation of acetylated p53 by inhibiting SIRT1 [32], the acetylation status of p53 was unchanged at 3 µM of tenovin-6, a concentration at which we observed the differentiation of NB4 cells (Figure 4B). Instead, upon tenovin-6 treatment, we observed an accumulation of acetylated α-tubulin, a target of SIRT2 (Figure 4B). We hypothesized that tenovin-6 induces granulocytic differentiation by inhibiting SIRT2 activity in NB4 cells.

### SIRT2 Knockdown Induces Granulocytic Differentiation in NB4 Cells

Although all evidence so far supports the idea that inhibition of sirtuins induces granulocytic differentiation in NB4 cells, we cannot exclude the possibility that tenovin-6 inhibits an enzyme(s) other than sirtuins to induce cellular differentiation. In addition, because it is not clear which sirtuin is responsible for the regulation of granulocytic differentiation, we examined the effect on granulocytic differentiation in NB4 cells when SIRT1 or SIRT2 was knocked down by shRNA.

**Figure 5:**
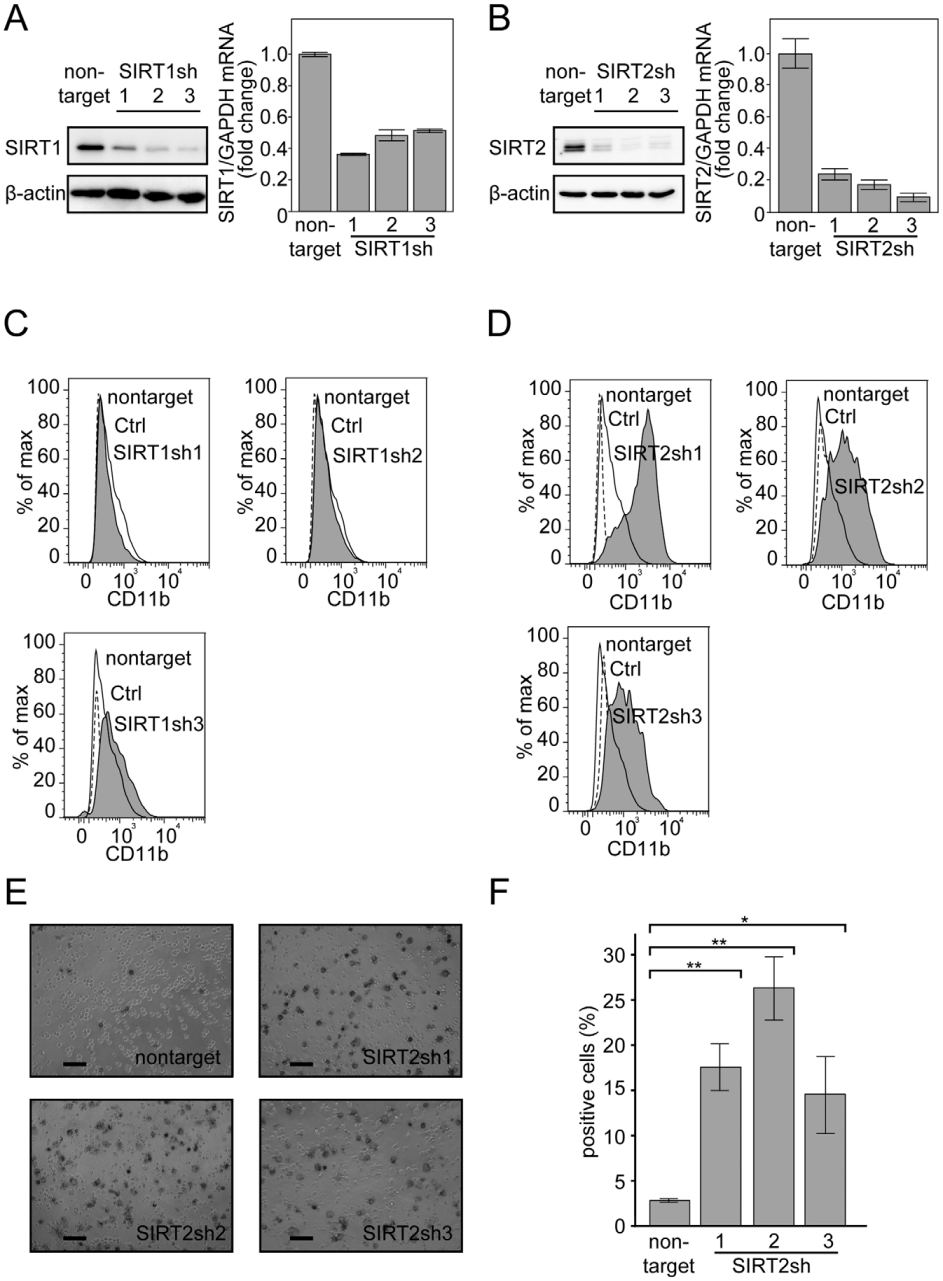
SIRT2 knockdown induces granulocytic differentiation in NB4 cells. (A, B) Knockdown efficiency was examined by immunoblotting and RT-PCR for SIRT1 (A) and SIRT2 (B). β-Actin and GAPDH were used as controls for immunoblotting and RT-PCR, respectively. 7.5 (A) or 18 µg (B) extracts were loaded in each lane for immunoblotting. Three independent sequences of shRNAs against each gene were used. The RT-PCR results were quantified by the comparative Ct method. Non-target control values were set as 1, and relative fold values are depicted in the graph. Representative RT-PCR data are shown. Experiments were performed in triplicate, and error bars show mean ± SD. (C, D) Granulocyte marker CD11b expression was monitored by FACS. SIRT1-knockdown (C) and SIRT2-knockdown (D) cells (gray shadow) were compared with control cells expressing non-targeting shRNA (black line). PE-conjugated IgG control experiments were performed and are indicated as a dashed line. (E) NBT reduction assays against SIRT2-knockdown cells and control cells (non-targeting shRNA) were performed. The captured images were 2 days after treatment. (F) Cells with NBT reduction capacity were counted and are depicted in the graph. Counting was performed as described in Figure 1F. Error bars show mean ± SD (n = 3). *P<0.05. **P<0.01. Note that although we performed serial infections of NB4 cells, the infection efficiency was approximately 70%, judging from the proportion of cells infected with the control virus bearing a GFP expression construct (not shown). The subsequent assay was performed without establishing a cell line using a selection marker in the viral vector. Therefore, the analyzed population contains cells not expressing the shRNAs, so the readings in these assays were under-valued due to contamination by cells not bearing shRNAs.

Lentiviral vector pLKO2.0 bearing non-targeting shRNA (negative control) or 3 independent shRNA sequences against *SIRT1* or *SIRT2* were generated (see Materials and methods). shRNA expression was induced by the infection of NB4 cells with these viruses, and cells were subsequently cultured and harvested for the following experiments. All 3 shRNAs against either *SIRT1* (Figure 5A) or *SIRT2* (Figure 5B) significantly reduced their target RNA levels and protein accumulation in the infected cells 48 hours after the last infection of these viruses.

SIRT1 or SIRT2 knockdown cells were stained with CD11b antibody and analyzed by FACS. As shown in Figure 5C–D, SIRT2, but not SIRT1, knockdown significantly increased CD11b expression, indicating that decreased SIRT2 expression is a prerequisite for granulocytic differentiation of NB4 cells. This finding was also confirmed by NBT reduction assays of granulocytic differentiation (Figure 5E–F).

### Overexpression of SIRT2 Inhibits the Tenovin-6 Induced Granulocytic Differentiation

**Figure 6:**
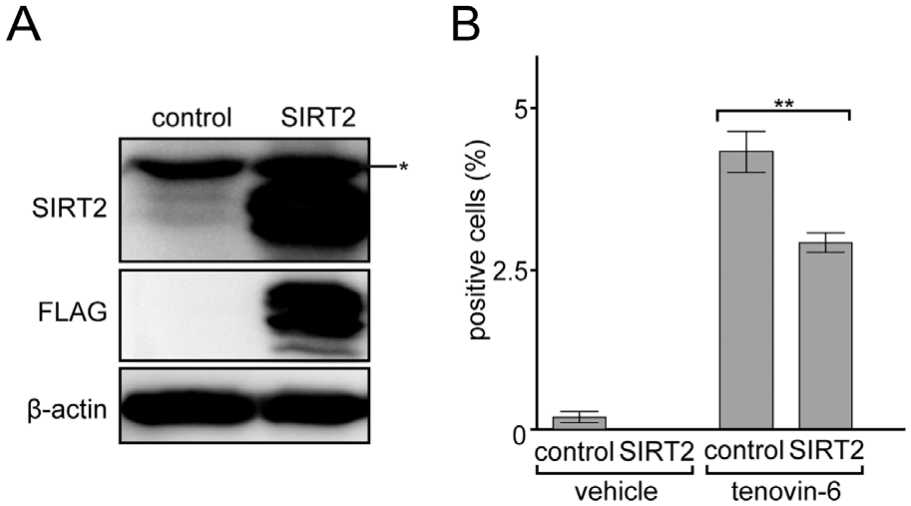
SIRT2 overexpression suppresses tenovin-6-dependent granulocytic differentiation in NB4 cells. (A) Immunoblot analysis for NB4 cells expressing FLAG-tagged SIRT2 or GFP (control). Extracts (56 µg) were loaded in each lane for immunoblotting by indicated antibodies. β-Actin was used as a control for immunoblotting. A single asterisk indicates the non-specific band. In the control lane, endogenous SIRT2 was detected as faint doublet bands. (B) NB4 cells were treated with 3 µM tenovin-6 or DMSO (vehicle) and cultured for 3 days, and the NBT-reduction capacity was monitored. NBT-positive cells were counted as described in Figure 1F. Error bars show mean ± SD (n = 3). **P<0.01.

Although we have shown that SIRT2 knockdown is sufficient for the granulocytic cell differentiation in NB4 cells, it is unclear whether tenovin-6 inhibits SIRT2 activity in the cells and whether the inhibition directly induces granulocytic cell differentiation. To assess the potential inhibition, we examined the effects of the overexpression of SIRT2 on the resistant of NB4 cells to tenovin-6 dependent granulocytic differentiation. We constructed a retrovirus expression vector bearing FLAG-tagged SIRT2 or GFP as a control. The packaged viruses were infected to NB4 cells, and cells expressing SIRT2 or GFP were obtained (Figure 6A) after the puromycin selection (details explained in the Materials and Methods section).

SIRT2-expressing or GFP-expressing (control) cells were treated with 3 µM tenovin-6 or DMSO (vehicle), and the NBT reduction capacity was examined. As shown in Figure 6B, both SIRT2-expressing and control cells treated with tenovin-6 exhibited increased NBT reduction capacity. However the number of NBT positive cells was lower in the SIRT2-expressing cells compared with the control. SIRT2 overexpression blocked granulocytic differentiation induced by tenovin-6, which indicates that tenovin-6 induces granulocytic differentiation at least in part, if not all by perturbing SIRT2 activity.

## Discussion

**Figure 7:**
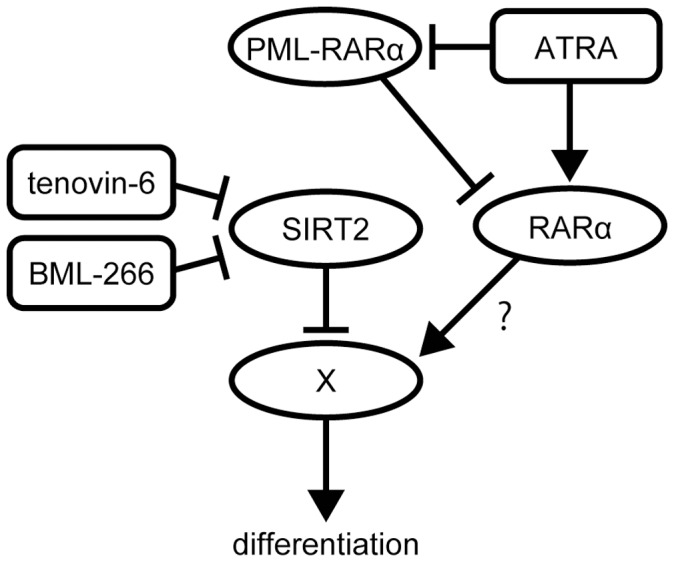
A hypothetical model for the induction of granulocyte differentiation by SIRT2 inhibition in leukemia cells. In NB4 cells, ATRA targets the PML-RAR-α oncogene product and promotes granulocytic differentiation. Inhibition of SIRT2 results in limited effects on PML-RAR-α accumulation and PML-NB formation, suggesting that the differentiation induced by the inhibition of SIRT2 is not due to an inhibition of PML-RAR-α but rather to the activation of a factor that plays a role in granulocytic differentiation whose activity is negatively regulated by deacetylation by SIRT2. In agreement with this, in PML-RAR-α-negative HL-60 cells, SIRT2 inhibition promotes granulocytic cell differentiation. Interestingly, ATRA also promotes cellular differentiation in HL-60 cells (data not shown) [54], suggesting that ATRA may target a pathway that is also targeted by SIRT2 inhibition.

We have demonstrated that sirtuin inhibitors tenovin-6 and BML-266 induce granulocytic differentiation in the APL cell line NB4 (Figures 1 and 4). The tenovin-6-induced differentiation is likely due to an inhibition of SIRT2 deacetylase activity, as we detected increased acetylated α-tubulin, a SIRT2 substrate (Figure 4). Furthermore, tenovin-6 induces granulocytic differentiation in the myeloblastic leukemia cell line HL-60, which lacks PML-RAR-α, suggesting that tenovin-6 promotes cellular differentiation without perturbing the function of the PML-RAR-α oncogene product (Figure 2). In agreement with this finding, while ATRA treatment diminishes PML-RAR-α in NB4 cells and subsequently promotes PML-NB formation, tenovin-6 exhibits a limited effect on PML-RAR-α accumulation in NB4 cells and on PML-NB formation (Figure 2). Collectively, these data indicate that tenovin-6 induces granulocytic differentiation by modulating a pathway downstream of RAR-α (Figure 7) (discussed further below). Knocking down SIRT2, but not SIRT1, by shRNA induces granulocytic differentiation in NB4 cells (Figure 5). The inhibition of SIRT2 activity is sufficient to induce granulocytic differentiation in NB4 cells. Finally, the overexpression of SIRT2 may partially decrease the level of granulocytic differentiation induced by tenovin-6 (Figure 6), which indicated that tenovin-6 induces the cellular differentiation by inhibiting SIRT2 activity. Therefore, we propose that targeting SIRT2, a protein thought to function as a tumor suppressor in cancer cells [26], [27], is a viable strategy for inducing leukemic cell differentiation.

ATRA induces granulocytic differentiation in APL cells both *in vitro* and *in vivo* by activating RAR-α in the PML-RAR-α fusion protein by forcing transcriptional repressor complexes to dissociate from RAR-α [3] and simultaneously degrading PML-RAR-α oncogene products (Figure 2) [40]. Because ATRA possesses a strong capacity to activate wild-type RAR-α and its downstream targets, ATRA promotes granulocytic differentiation in other leukemia cells, even those lacking a PML-RAR-α fusion protein. [41] Similarly, tenovin-6 promotes granulocytic differentiation in not only the APL cell line NB4 (PML-RAR-α positive) but also the myeloblastic leukemia cell line HL-60 (PML-RAR-α negative). These facts raise 2 possibilities to explain the mechanism of action of tenovin-6 in granulocytic differentiation: (a) similar to ATRA, tenovin-6 activates RAR-α regardless of whether it is fused to PML; or (b) tenovin-6 modulates the pathway downstream of RAR-α to mimic RAR-α activation.

Although tenovin-6 treatment induces a partial decrease of PML-RAR-α protein in NB4 cells (Figure 2), we did not observe any significant alteration in PML-NB formation in tenovin-6-treated cells. This result suggests that in tenovin-6-treated cells, there are still sufficient numbers of PML-RAR-α proteins to block wild-type PML protein from forming PML-NBs and, presumably, to block RAR-α function in transcriptional regulation. Therefore, SIRT2 likely modulates the downstream components of the RAR-α pathway to induce granulocytic differentiation in leukemia cells (Figure 7). However, SIRT2 perturbation might modulate transcriptional repressors bound to PML-RAR-α, subsequently leading to the activation of RAR-α to promote cellular differentiation. Further characterization of SIRT2-knockdown cells and tenovin-6-treated cells in terms of RAR-α activation is required to assess this possibility.

Both tenovin-6 and ATRA induce granulocytic differentiation in PML-RAR-α-negative cells, which raises the possibility that ATRA downregulates SIRT2 deacetylase activity to induce cellular differentiation. To assess this possibility, we examined the acetylation status of the SIRT1 and SIRT2 substrates p53 and α-tubulin, respectively, but we did not observe any significant alteration of either protein (data not shown). This result suggests that ATRA does not directly perturb SIRT1 and SIRT2 activity to induce granulocytic differentiation. Interestingly, proteomic analysis revealed that 14-3-3η and 14-3-3ζ/δ are significantly decreased after ATRA treatment [42]. 14-3-3ζ serves as a suppressor of transcription factors such as FOXO3A [43], and the interaction between 14-3-3ζ and caspase 2 can be positively regulated through sirtuin deacetylase activity [44]. Therefore, it is conceivable that either inhibition of sirtuins or degradation of 14-3-3 leads to activation of the pathway that is negatively regulated by 14-3-3 binding and sirtuin deacetylase activity. The granulocytic differentiation observed either by the inhibition of SIRT2 or ATRA treatment may be caused by a modulation of the same pathway (Figure 7). More detailed analyses of 14-3-3, sirtuins, and their presumptive common targets are required to test this hypothesis.

In rodents, SIRT2 is defined as a tumor suppressor by maintaining genome stability; however, recent studies show that SIRT2 can be a therapeutic target in cancer treatment. In primary AML, the levels of SIRT2 mRNA and protein in CD34^+^ cells are upregulated compared with cells from healthy individuals [31]. The inhibition of SIRT2 by a SIRT2 inhibitor AC93253 induces apoptosis selectively to AML cells [31]. In addition, in vertebrate, SIRT2-deficient cells are susceptible to apoptosis when treated with anti-cancer reagents such as staurosporine or cisplatin [45]. The observations imply that SIRT2 possesses anti-apoptotic and oncogenic properties perhaps by the inhibition of Foxo1, an apoptosis regulatory factor [46], [47]. The contradictory evidence about the role of SIRT2 in tumorigenesis may be due to the diverse functions of SIRT2. Nevertheless, our observation suggests that SIRT2 possesses an oncogenic role by blocking cellular differentiation at least in APL.

Although tenovin-6 demonstrated a strong cytotoxic effect against NB4 cells at a higher concentration (Figure 1), SIRT1- or SIRT2-knockdown cells did not immediately die, except for SIRT1-sh3 cells (data not shown). Because the 2 other SIRT1 shRNAs did not exhibit strong cytotoxic effects, the SIRT1-sh3 effect was likely due to an off-target effect. This observation suggests that knocking down SIRT1 is not sufficient to induce cell death in APL cells. Nevertheless, previous reports illustrate the importance of targeting SIRT1 in other cancer cells [22], [32]. Although our results strongly suggest that tenovin-6 directly target SIRT2 to induce cellular differentiation in NB4 cells (Figure 6), tenovin-6 may target other enzymes to induce cellular death and/or differentiation. Tenovin-6 shows relatively weak inhibition of SIRT3 protein deacetylase activity [32] which may play a role in the efficacy of tenovin-6.

In summary, we have identified SIRT2 as a potential therapeutic target in APL treatment. Because of the limited number of primary APL cells, we were so far unable to demonstrate tenovin-6-dependent cell differentiation with primary cells. However, tenovin-6 shows cytotoxicity against APL primary cells (not shown), which indicates its potential as a therapeutic compound against APL. Several different sirtuin inhibitors including tenovin-6 have been shown to be effective in the treatment of hematologic malignancies in mouse models [34], [48]. Therefore, further studies using relevant mouse models should lead to the development of new therapeutic approaches targeting SIRT2 to overcome ATRA resistance in APL patients.

## Materials and Methods

### Cell Culture and Proliferation Assay

NB4 [49] and HL-60 (obtained from American Type Culture Collection) cells were cultured in IMDM medium (Invitrogen) with 10% heat-inactivated fetal calf serum (HyClone), 100 U/mL penicillin, and 100 µg/mL streptomycin at 37°C in a humidified 5% CO_2_ atmosphere. 293T cells were cultured in DMEM (Nacalai Tesque) supplemented with 10% fetal calf serum, 100 U/mL penicillin, and 100 µg/mL streptomycin (Sigma-Aldrich) at 37°C in a humidified 5% CO_2_ atmosphere. Cell proliferation (Figure 1A) was evaluated with a CellTiter 96 Non-Radioactive Cell Proliferation Assay kit (Promega) following the manufacturer’s protocol. Briefly, 1×10^4^ cells were seeded in a 96-well plate with 100 µL medium, cultured for 48 hours in the presence of tenovin-6 or DMSO (vehicle), and mixed with MTT solution; absorbance at 570 nm was then measured using a Model 680 Microplate Reader (Bio-Rad). Cell proliferation and viability (Figures 1C and 3A) were measured by the trypan-blue dye exclusion assay using a TC10 Automated Cell Counter (Bio-Rad).

### Compounds

ATRA (Sigma #R2625) was dissolved in absolute ethanol at 1 mM. Tenovin-6 (Cayman Chemical #13086) and BML266 (Santa Cruz #sc-221371) were dissolved in dimethyl sulfoxide (DMSO) at 10 mM and 50 mM, respectively. Dissolved compounds were stored at –20°C untill use.

### Cell Morphologic Analysis and NBT Reduction Assays

To examine cellular morphology, cells were cytospun, stained with Wright-Giemsa staining solution, and observed under a BX51 microscope (Olympus). Granulocytic differentiation was monitored by a nitroblue tetrazolium (NBT) reduction assay as described previously [50]. Briefly, cells were incubated with a 1∶1 mixture of culture medium and PBS containing 50 µg/mL Nitro-TB (Dojinbo) and 0.1 µg/mL phorbol 12-myristate 13-acetate (Sigma-Aldrich) at 37°C in a humidified 5% CO_2_ atmosphere for 1 hour, and then images of cells were captured using a Biozero-8100 microscope (KEYENCE).

### Flow Cytometry

To monitor apoptosis induced by tenovin-6, an Annexin-V-PI assay was performed with a CELL LAB ApoScreen Annexin V-FITC Apoptosis Kit (Beckman coulter) on a FACScan (BD Biosciences) by following the manufacturer’s protocol. To examine APL cell differentiation, cells were stained with phycoerythrin (PE)-conjugated anti-CD11b antibody (Beckman coulter, IM2581U) and were then analyzed in a Navios Flow Cytometer (Beckman Coulter). The 7-AAD dye (Beckman Coulter, A07704) was used to stain and exclude dead cells. The obtained data were further analyzed with FlowJo software (Tree Star).

### PML-NB Visualization by Immunofluorescence Microscopy

Tenovin-6- or DMSO-treated NB4 cells were cytospun on MAS-coated glass slides (Matsunami Glass) at 30 *g* for 5 minutes using a Cyto-Tek Centrifuge (SAKURA), washed with PBS, and fixed with PBS containing 4% freshly prepared paraformaldehyde on ice for 10 minutes. Cells were treated with washing buffer (0.1% Triton X-100/PBS), incubated with blocking buffer (5% horse serum/0.3% Triton X-100/0.1% sodium azide/PBS) on ice for 1 hour, and then 1∶50-diluted anti-PML antibody (Santa Cruz, PG-M3) in blocking buffer at 4°C overnight. After incubation with the primary antibody, cells were rinsed and incubated with the washing buffer on ice for 3×5 minutes and were then incubated with 1∶400-diluted Alexa Fluor 488-conjugated goat anti-mouse IgG (Life Technologies) in blocking buffer on ice for 2 hours. After rinsing and incubation with washing buffer on ice for 3×5 minutes, cells were mounted in ProLong Gold Antifade Reagent (Life Technologies) containing DAPI (4,6 diamidino-2-phenylindole) and viewed under a TCSSP5 laser confocal microscope (Leica).

### Immunoblotting

To make cell extracts for immunoblotting, cells were washed with PBS containing 10 mM nicotinamide, 1 µM trichostatin A (TSA), 1 mM orthovanadate, and 20 mM sodium fluoride and were then sonicated in RIPA buffer (Cell Signaling) supplemented with 10 mM nicotinamide, 1 µM TSA, 1 mM orthovanadate, 20 mM sodium fluoride, 1 µg/mL aprotinin (Sigma-Aldrich), 2 µg/mL E-64 (Roche Applied Science), 1 µg/mL leupeptin (Roche Applied Science), 0.67 µg/mL bestatin (Calbiochem), 0.67 µg/mL pepstatin (Roche Applied Science), and 43.5 µg/mL PMSF (Sigma-Aldrich) [51]. To detect PML-RAR-α, a urea-containing buffer [6] was used to make cell extracts because of the insolubility of PML-RAR-α protein in RIPA buffer. The protein concentration was determined by the BCA protein assay kit (Thermo Scientific). Equal amounts of protein was denatured, electrophoresed, and blotted. The following primary antibodies were used for immunoblotting detection: anti-RAR-α, (Santa Cruz #sc-551), anti-acetyl-Lys382p53 (Cell Signaling #2525), anti-p53 (Cell Signaling #9252), anti-acetyl-Lys40α-tubulin (Cell Signaling #3971), anti-tubulin (Sigma-Aldrich #T5168), anti-SIRT1 (Sigma-Aldrich #S5322), anti-SIRT2 (Abgent #AJ1718a; Figure 5), anti-SIRT2 (Santa Cruz #sc-20966; Figure 6), and anti-β-actin (Cell Signaling #4967). The following horseradish peroxidase-conjugated secondary antibodies were used: polyclonal rabbit anti-mouse IgG (DAKO #Z0259) for anti-tubulin and polyclonal swine anti-rabbit IgG (DAKO #Z0196) or goat anti-rabbit IgG (Santa Cruz #sc-2004) for other primary antibodies. The chemiluminescence reaction was performed with Pierce ECL Western Blotting Femto (Thermo Scientific), and images were captured using an LAS-3000 or LAS-4000 (Fuji).

### Lentiviral shRNA Expression

To perform knockdown experiments, the following shRNA sequences obtained from the RNAi consortium database (Broad Institute) were subcloned into the AgeI and EcoRI sites of lentiviral vector pLKO2.0. Non-targeting sh: CCGGCAACAAGATGAAGAGCACCAACTCGAGTTGGTGCTCTTCATCTTGTTGTTTTTG; SIRT1sh1: CCGGGCAAAGCCTTTCTGAATCTATCTCGAGATAGATTCAGAAAGGCTTTGCTTTTTG.

AATT; SIRT1sh2: CCGGCCTCGAACAATTCTTAAAGATCTCGAGATCTTTAAGAATTGTTCGAGGTTTTTG.

AATT; SIRT1sh3: CCGGGCGGGAATCCAAAGGATAATTCTCGAGAATTATCCTTTGGATTCCCGCTTTTTG.

AATT; SIRT2sh1: CCGGGCCATCTTTGAGATCAGCTATCTCGAGATAGCTGATCTCAAAGATGGCTTTTTGAATT; SIRT2sh2: CCGGGCTAAGCTGGATGAAAGAGAACTCGAGTTCTCTTTCATCCAGCTTAGCTTTTTGAATT; and SIRT2sh3: CCGGTATGACAACCTAGAGAAGTACCTCGAGGTACTTCTCTAGGTTGTCATATTTTTGAATT. To produce lentivirus particles, sequence-verified plasmids were transfected into 293T cells using polyethylenimine (Polysciences) [52] with the helper vectors pCMVR8.91 and MD2G (kind gift from Dr. Didier Trono). Forty-eight hours later, the supernatant containing lentivirus particles was collected and then concentrated by centrifugal filter devices (Amicon Ultra-15, Millipore). Approximately 500,000 cells were infected 4 times with concentrated virus every 12 hours and were subsequently cultured for assays.

### Real-time Quantitative PCR

Total RNA was isolated from cells with the PureLink RNA Mini Kit (Life Technologies). cDNA was synthesized from 250 to 350 ng RNA with the ReverTra Ace qPCR RT Kit (TOYOBO) following the manufacturer’s protocol. Synthesized cDNA (2 µL) was analyzed by RT-PCR performed with THUNDERBIRD SYBR qPCR Mix (Toyobo) in a CFX96 Real-Time system (Bio-Rad). The following primers were used for RT-PCR: *SIRT1* (forward: AAATGCTGGCCTAATAGAGTGG, reverse: TGGTGGCAAAAACAGATACTGA); *SIRT2* (forward: GAACGCTGTCGCAGAGTCATC, reverse: GGTTGGCTTGAACTGCCCAG); and *GAPDH* (forward: AGCCACATCGCTCAGACAC, reverse: GCCCAATACGACCAAATCC). Quantification was performed by the relative standard curve method against the control *GAPDH*.

### Retroviral SIRT2 Overexpression

Carboxy-terminal FLAG-tagged *SIRT2* cDNA (Addgene #13813) [53] was excised by PmeI and subcloned into a blunt-ended EcoRI site in a retro-viral vector pQCXIP (Promega). As a control vector, turbo-GFP cDNA was PCR-amplified from pLKO1-turboGFP (Sigma) and subcloned into a NotI site in pQCXIP. To produce retrovirus particles, sequence-verified plasmids were transfected into 293T cells using polyethylenimine (Polysciences #23966) [52] with the helper vectors pCL10A1 (Imgenex). The virus infection was performed as described in the “Lentiviral shRNA expression” section. The selection for SIRT2-FLAG- or GFP-expressing cells was performed by 2 µg/ml puromycin containing media.

### Statistical Analysis

Levels of significance for comparison between samples were determined by the Student *t-*test. P<0.05 was considered statistically significant. All the experiments were performed at least 3 times.
