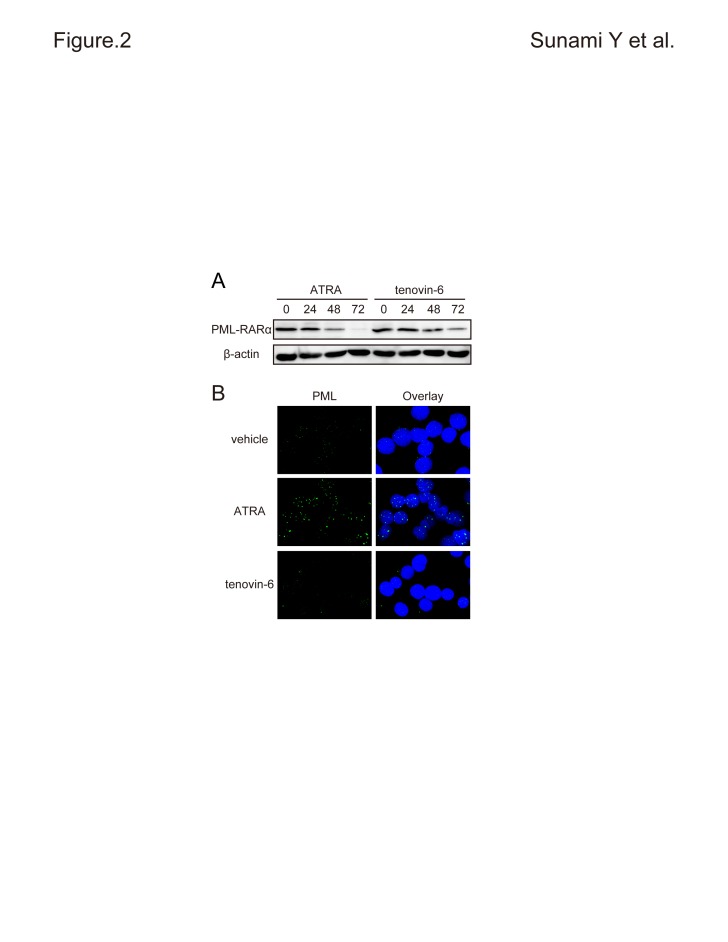# Correction: Inhibition of the NAD-Dependent Protein Deacetylase SIRT2 Induces Granulocytic Differentiation in Human Leukemia Cells

**DOI:** 10.1371/annotation/36fa9e18-5e99-466d-b10d-dc67cfd14682

**Published:** 2013-11-06

**Authors:** Yoshitaka Sunami, Marito Araki, Yumi Hironaka, Soji Morishita, Masaki Kobayashi, Ei Leen Liew, Yoko Edahiro, Miyuki Tsutsui, Akimichi Ohsaka, Norio Komatsu

The lower left panel of Figure 2, PML staining for tenovin-6, appears incorrectly as a duplicate of the upper left panel. Please see the corrected Figure 2 here: 

**Figure:**